# i2dash: Creation of Flexible, Interactive, and Web-based Dashboards for Visualization of Omics Data

**DOI:** 10.1016/j.gpb.2021.01.007

**Published:** 2021-07-17

**Authors:** Arsenij Ustjanzew, Jens Preussner, Mette Bentsen, Carsten Kuenne, Mario Looso

**Affiliations:** Max Planck Institute for Heart and Lung Research, Bioinformatics Unit, D-61231 Bad Nauheim, Germany

**Keywords:** Data visualization, Dashboard, Cloud, Data analysis, Automation

## Abstract

**Data visualization** and interactive data exploration are important aspects of illustrating complex concepts and results from analyses of omics data. A suitable visualization has to be intuitive and accessible. Web-based **dashboards** have become popular tools for the arrangement, consolidation, and display of such visualizations. However, the combination of automated data processing pipelines handling omics data and dynamically generated, interactive dashboards is poorly solved. Here, we present i2dash, an R package intended to encapsulate functionality for the programmatic creation of customized dashboards. It supports interactive and responsive (linked) visualizations across a set of predefined graphical layouts. i2dash addresses the needs of data analysts/software developers for a tool that is compatible and attachable to any R-based analysis pipeline, thereby fostering the separation of data visualization on one hand and **data analysis** tasks on the other hand. In addition, the generic design of i2dash enables the development of modular extensions for specific needs. As a proof of principle, we provide an extension of i2dash optimized for single-cell RNA sequencing analysis, supporting the creation of dashboards for the visualization needs of such experiments. Equipped with these features, i2dash is suitable for extensive use in large-scale sequencing/bioinformatics facilities. Along this line, we provide i2dash as a containerized solution, enabling a straightforward large-scale deployment and sharing of dashboards using **cloud** services. i2dash is freely available via the R package archive CRAN (https://CRAN.R-project.org/package=i2dash).

## Introduction

Interactive data visualization is of vital importance when dealing with complex or extensive data as regularly produced in the fields of physics, geography, or life sciences. In this context, recently published software packages providing interactive data visualizations in a web browser, such as Shiny (https://shiny.rstudio.com/) and Plotly [1], have become popular. Many recently developed software tools utilizing these “meta-level” packages were introduced. For instance, when focusing on the R programming environment in the field of life sciences, the WIlsON [2] package represents an example of a generic omics data viewer with a collection of flexible modules for interactive visualization and data handling functionalities. However, most packages are intended for the examination of one specific type of (omics) data, which enables highly adapted functionality and tailored data views, but with limited flexibility in regards to extensibility. In addition, most of these tools rely on and are restricted to a distinct data object in a certain format, *e.g.*, the complex R SingleCellExperiment object [3]. Although such format dependencies ensure the high performance of the visualization tools, they limit computational biologists to predefine plots, types of visualization, and fixed layouts. A generic software package that can be integrated into any kind of data generation process (such as an omics data analysis pipeline), enabling a flexible, web-based data presentation and exploration functionality and featuring custom layouts, is critically missing. Here we introduce i2dash, a tool that provides functionality for data analysts and software developers working with any kind of omics data using complex workflows. i2dash enables the programmatic creation of interactive dashboards from within such workflows, reduces code complexity, and simplifies the spatial arrangement of data visualizations.

**Figure 1:**
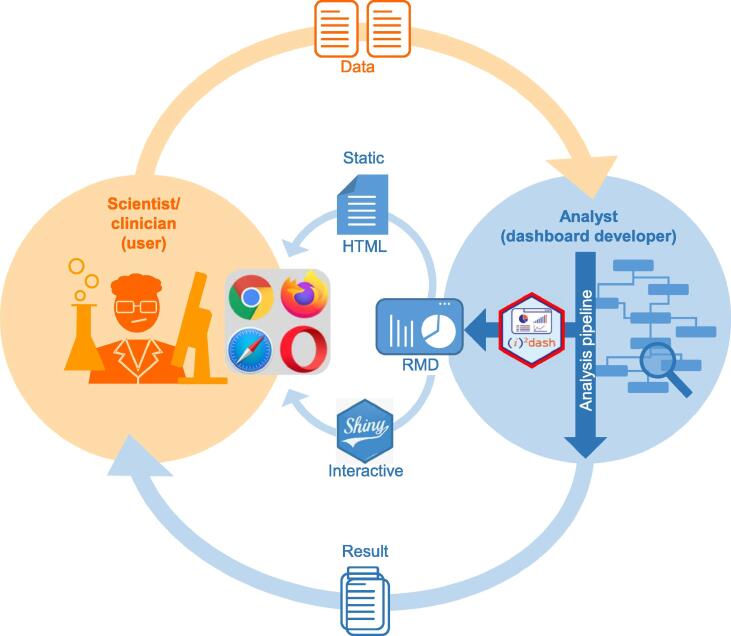
**Operational interface of i2dash** Users and developers (*e.g.*, computational analysts) are typically connected by process of data generation, data analysis, provisioning of results, and an iterative process of analysis refinement (outer orange and blue arrows). i2dash (red hexagon) supports this process by integrating any kind of R-based analysis pipeline or software (blue circle, right) and enables the developer to provide a dynamic data interpretation/data view, organized as an interactive dashboard. i2dash provides functionality to generate both a static dashboard based on HTML or an interactive dashboard based on R Shiny (inner blue arrows). Utilizing these web technologies, technical hurdles of distribution and access are minimized, and users only depend on a web browser of their choice (orange circle, left).

In this context, i2dash is intended to primarily act at the interface of non-computational users (NCU) and computational analysts and developers (CAD) (Figure 1). It provides functionality to generate dashboard-based data views for CADs working with any kind of omics data. i2dash is designed to provide dashboards both as HTML websites and interactive Shiny apps. Since the latter requires at least a local server or an advanced IT infrastructure for even larger deployments (*e.g.*, in cloud environments), we provide a cloud-compatible docker container to assist CADs with dashboard distribution (see Method). In addition to this functionality, i2dash is designed to work with custom extensions for specific data types in order to generate adapted visualization venues in research and industry. As a proof of principle, we developed a single-cell RNA sequencing (scRNA-seq) extension, enabling the generation of dashboards optimized for a typical single-cell (SC) data analysis workflow with very few function calls, providing interlinked visualizations among others (see the “i2dash.scrnaseq extension” section below). Further, we benchmark our extension to a collection of SC specific visualization tools that create static HTML reports for quality control and data set comparisons, such as scRNABatchQC [4], or interactive R/Shiny-based applications that allow data exploration and visualization, such as Cerebro [5], iSEE [6], and pcaExplorer [7]. As shown in the comparison for this use-case example, there is no need to define the final layout for an i2dash dashboard at the beginning of an analysis workflow. Instead, i2dash acts on a visualization meta-level, providing an iterative and dynamic process of dashboard creation, started at runtime.

In summary, i2dash liberates CADs from the struggle with low-level code or time-consuming spatial arrangement of data visualizations when generating data views tailored to the respective audience. From an NCU's point of view (Figure 1), i2dash provides a simplified access to high-dimensional data interpretations via a web browser with a focus on simple provisioning and low technical hurdles. Hence, i2dash is the first choice to supersede static reports by state-of-the-art responsive, interactive, and flexible dashboards. Along this line, i2dash provides the optimal framework for tool developers who want to summarize their results in an easy-to-use, web-accessible format.

## Method

**Figure 2:**
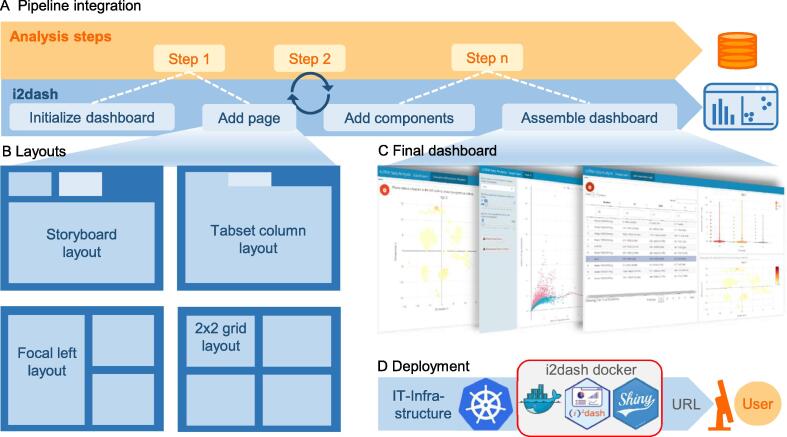
**Pipeline integration and i2dash mechanism of action** **A.** i2dash is integrated at the beginning of a data analysis pipeline (orange arrow) by initialization of an empty dashboard (blue arrow). During the pipeline run, new content, results, or data visualizations are iteratively added to the dashboard’s pages. The final dashboard is rendered into a static or interactive document. **B.** Pages added to the dashboard can be arranged in a flexible manner from a selection of predefined layouts. **C.** Examples of programmatically created dashboards. **D.** The i2dash docker container, providing all dependencies for interactive Shiny-based apps, can be used to deploy individual data interpretations on a cloud infrastructure (*e.g.*, Kubernetes) as a microservice.

The typical workflow for the creation of a dashboard with i2dash is a flexible process, suitable for integration into existing analysis pipelines, that generates results iteratively (Figure 2A). Once an instance of a dashboard has been initialized, CADs can add pages with various layouts (Figure 2B), enabling different content presentation strategies (Table 1). Individual components such as texts, images, tables, and interactive widgets can be added to pages as defined by the inherent layout (Table 1). Noteworthy, it is possible to link components into responsive views, such that data points visualized and selected/filtered for in one component can trigger a change in the other. Dashboard creation concludes with the assembly of an RMarkdown document from the i2dashboard instance, which can be directly rendered into a static HTML file (*e.g.*, for report generation, archiving, or sharing with colleagues) or into an interactive Shiny app running on the local computer for web-based exploration (Figure 2C). The integration of dashboards into professional service facilities requires more advanced infrastructure features. Such advanced setups often deal with recurring analysis tasks, application virtualization, and cloud computing infrastructure, a scenario that is supported by i2dash. Accordingly, we provide a docker container for the deployment of interactive dashboards on local servers or even cloud infrastructure platforms such as Kubernetes (Figure 2D). In a typical use case, a service facility provides a running instance of the interactive dashboard to collaborators for interactive exploration of their data analysis. Following the FAIR data principles, access to the interactive dashboard can additionally be granted to the public audience, *e.g.*, as an accompanying supplementary resource upon publication (for a set of example use cases as described in the “i2dash.scrnaseq extension” section below, see https://loosolab.github.io/i2dash.scrnaseq/#use-cases).

**Table 1:** **Available dashboard layouts and features**

**Layout**	**Feature**	**Useful for**
Storyboard	Unlimited capacity	Presenting a sequence of data visualizations and related commentary
Tabset column	Unlimited capacity	Presenting a large number of components without requiring the user to scroll
Focal left	Up to three components linkable layout	Highlighting a single component
2 × 2 grid	Up to four components linkable layout	Cross-linked components / interactivity
Sidebar	Globally or locally	Providing static content and UI elements to control interactive components

## Implementation

**Figure 3:**
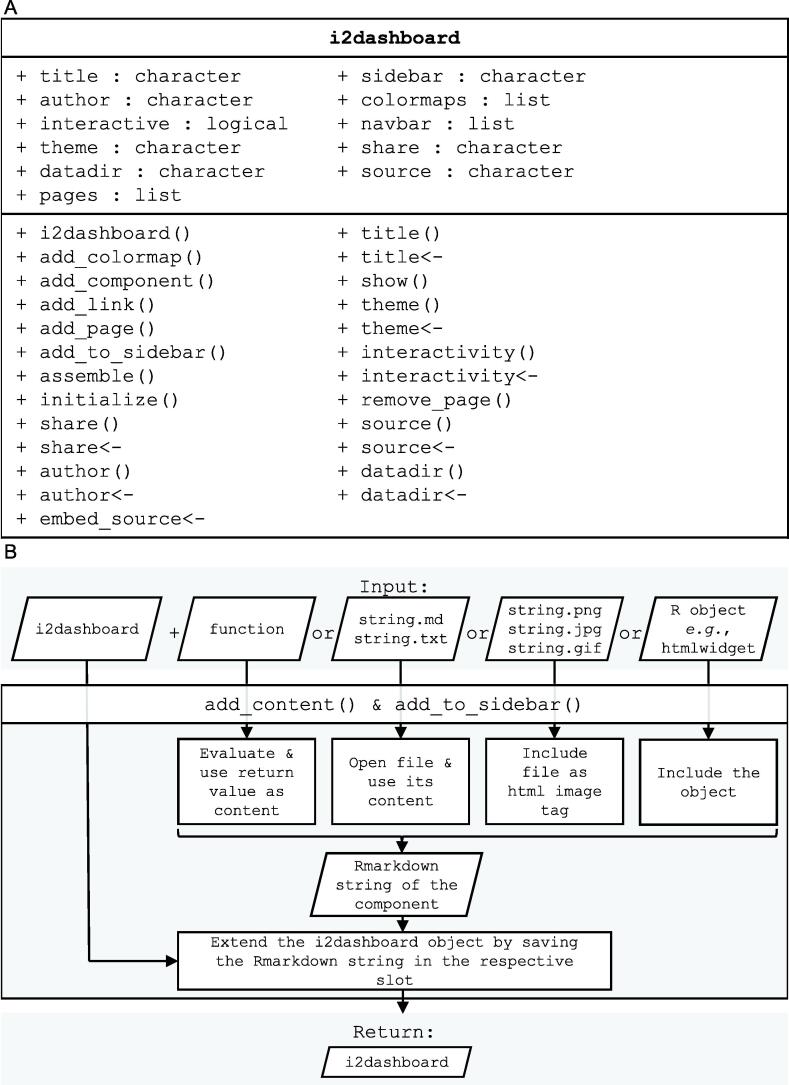
**The i2dashboard class and package organization** **A.** The i2dashboard class is based on the S4 object system. It stores global properties such as the dashboard title, author, and theme (upper left), as well as individual dashboard pages, sidebars, navigation bar items, and document-wide colormaps (upper right). The class provides a versatile list of functions to initiate, assemble, and add content to a dashboard (lower panel). **B.** i2dash leverages generic functions to dispatch arguments based on their individual class(es). The same generic function might receive an image, a file path, an object of a supported HTML widget class, or even a function as arguments (upper boxes), but exhibit different mechanisms to create content (middle). In all cases, the plain RMarkdown code is returned and is added to the dashboard upon assembly (lower boxes). The devised mechanism allows the design of templates that add ready-made visualizations, layouts, and interactive tools to dashboards.

The R package i2dash is entirely written in the R programming language and relies on the widely used R packages flexdashboard (https://CRAN.R-project.org/package=flexdashboard), knitr [8], RMarkdown [9], and stringi, and suggests ComplexHeatmap [10], ggplot2 [11], and switchr [12] as well as biocstyle [13]. i2dash introduces a new class based on R’s S4 object system named i2dashboard (Figure 3A), which by design provides the main functionality of the package. Besides global properties such as the dashboard title, author, and theme, an instance of the i2dashboard class stores individual dashboard pages, sidebars, navigation bar items, and document-wide colormaps, as well as all components that make up the content of individual pages.

We provided i2dash with a versatile mechanism to add content to a dashboard (Figure 3B). Its generic functions can dispatch arguments based on their individual class(es). For example, the same generic function might add an image or an HTML widget to the dashboard, depending on whether it gets passed a file path or an object of a supported HTML widget class. This feature allows i2dash to handle objects of more than 50 different classes (for a complete list, see gallery.htmlwidgets.org). Moreover, i2dash implements the ability to pass functions as an argument to generic functions that are only expected to return plain RMarkdown code upon execution. Those functions allow the injection of arbitrary and user-defined content into the dashboard, constituting the most flexible mechanism conceivable.

Aside from the generation of highly flexible dashboards, the ability to pass functions as arguments to generic functions permits the design of templates that add ready-made visualizations, layouts, and interactive tools to dashboards. As a proof of principle, we developed an extension of i2dash as another R package for the exploration of results from scRNA-seq called i2dash.scrnaseq (see the “i2dash.scrnaseq extension” section below). Along this line, i2dash.scrnaseq can be seen as a template to shed light on certain aspects of scRNA-seq data generated and inspired by an individual CAD. Once generated, this view is shareable and permanently available.

## i2dash.scrnaseq extension

**Figure 4:**
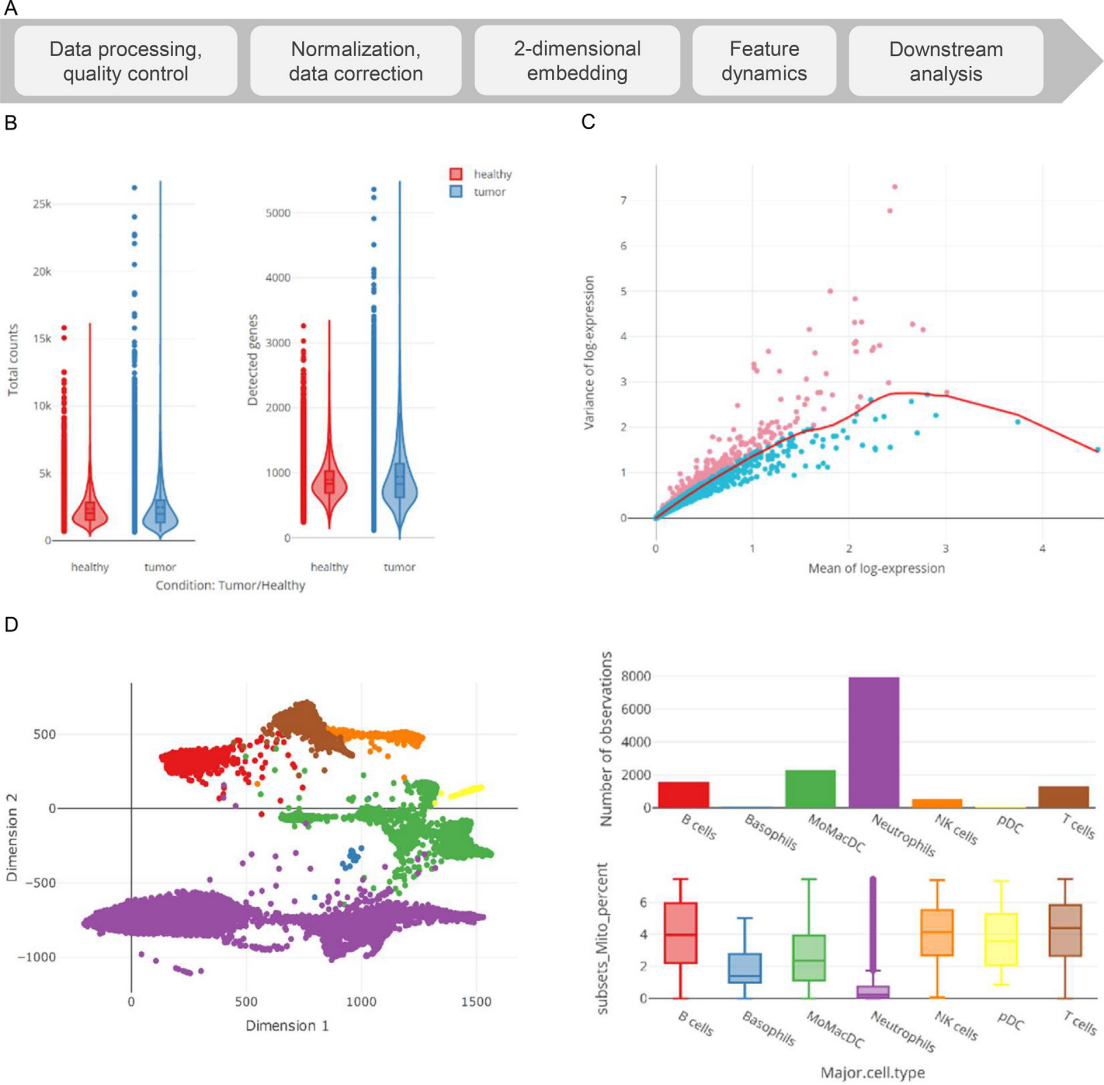
**Visualization features of the i2dash.scrnaseq extension** **A.** High-level workflow for a typical analysis of data from a scRNA-seq experiment. **B.** Violin plots from the plotly package showing per-cell quality criteria, such as the number of read counts (left) and the number of detected genes (right). Plots are stratified by the categorical variable “Condition”. **C.** Visualization of expression mean and variance of features (mean–variance plot) generated by the “Feature selection page”. Highly variable features are highlighted in red and can be used for downstream analysis. **D.** Exploration of cell metadata using linked visualizations. Individual samples are shown in a 2D embedding (left), colored by their annotated cell type. Upon sample selection, the number of observations per cell type (top right) and the percentage of mitochondrial content across cell types (bottom right) are updated, respectively. scRNA-seq, single-cell RNA sequencing.

As indicated above, i2dash.scRNAseq is an illustrative example for an extension of i2dash, suitable for the generation of specialized data views (Table S1). It is an individual solution for advanced visualization of transcriptomics data and is designed to assist with typical steps performed during the analysis of data from scRNA‐seq. In order to showcase the functionality of this custom extension, we analyzed a previously published dataset ([14], GSE127465) of human and mouse lung cancers with scater [15], following the current best practices in scRNA-seq analysis [16] (Figure 4A), and populated an interactive dashboard using i2dash.scrnaseq with typical views for this kind of data (Table S2). The data shown below are interactively accessible in three use cases from https://loosolab.github.io/i2dash.scrnaseq/#use-cases.

Starting with data quality control (Figure 4A, left), i2dash.scrnaseq provides boilerplate functions for boxplots, barplots, or violin plots, *e.g.*, to visualize the number of detected genes per cell (Figure 4B, use case 1 “Quality Metrics”). Optionally, those plots can be stratified by categorical variables (*e.g.*, sequencing batch) to investigate whether data correction is needed (Figure 4B). Per-cell quality data can additionally be aggregated across levels of categorical data (*e.g.*, to obtain the mean number of genes detected by sequencing batch) using any function to summarize numerical data (*e.g.*, mean, median, and sum). Feature selection, *e.g.*, using highly variable genes, can be visualized using a mean–variance plot (Figure 4C, use case 3 “Visual parameter selection for experts”). This plot is provided by adding the “Feature selection page” to a dashboard, which takes either a SingleCellExperiment object or a Seurat object [17] as input and additionally allows the user to choose a suitable model from scran [18].

**Figure 5:**
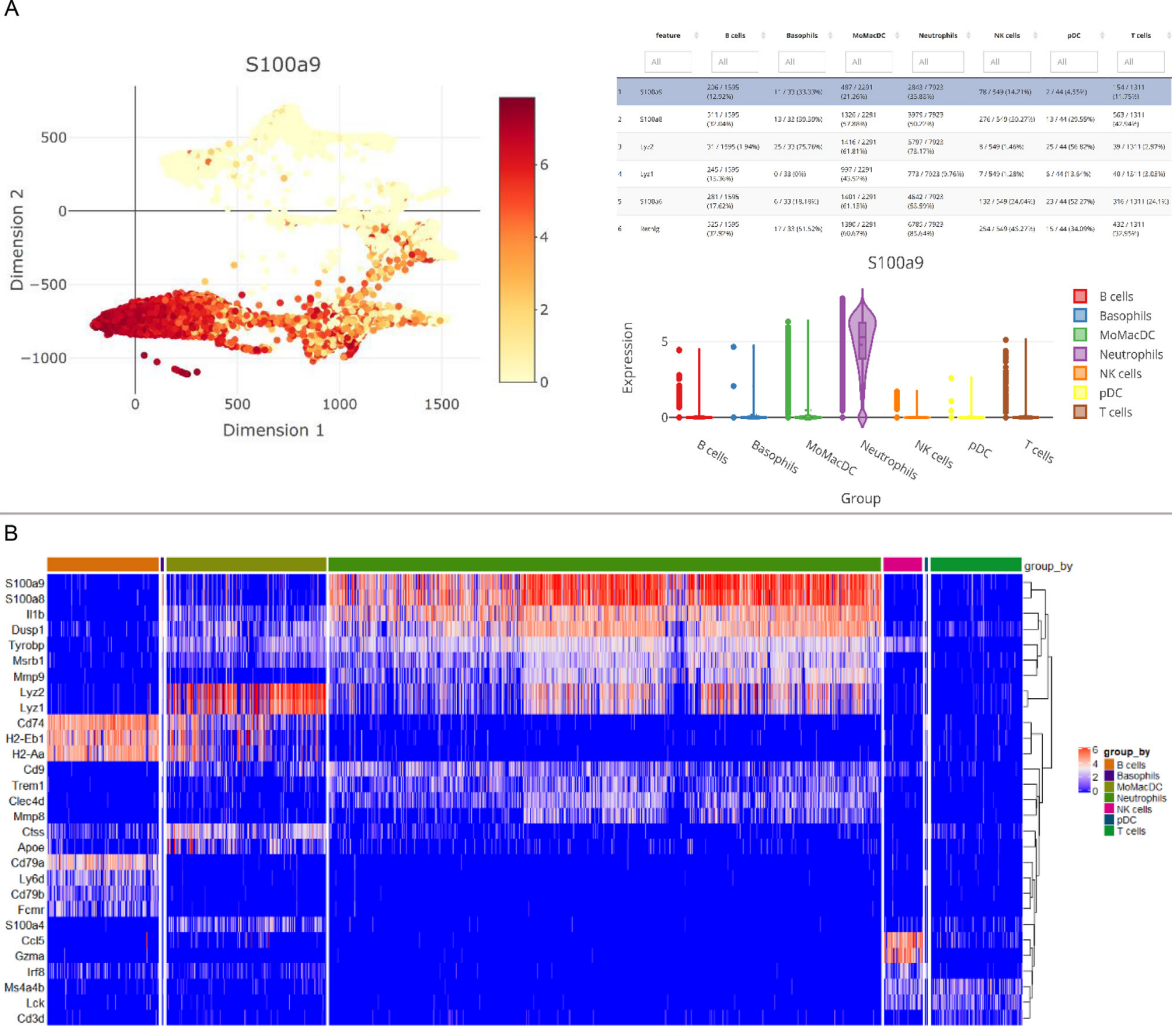
**Visualization of feature metadata and dynamics** **A.** Exploration of feature metadata using linked visualizations. Feature expression of individual samples is shown in a 2D embedding (left). A table (top right) shows features of interest (*e.g.*, from differential expression) and can update the coloring of the 2D embedding, as well as violin plots showing feature expression across cell types (bottom right). **B.** A heatmap is utilized to illustrate the expression dynamics of the top 10 marker genes for the annotated cell type.

Downstream analyses like cell clustering are often based on results from dimension reduction methods [*e.g.*, t-distributed stochastic neighbor embedding (t-SNE) or uniform manifold approximation and projection (UMAP)]. Consequently, high-level functions from i2dash.scrnaseq center around 2D embeddings (use case 2 “SC data explorer”), allowing the exploration of metadata either on sample level (*e.g.*, the number of cells per inferred cell type, Figure 4D) or feature level (*e.g.*, the expression of a marker gene in cell clusters, Figure 5A). By adding the “DimRed feature page” to a dashboard, the user can additionally display results from differential expression analysis in a table that is linked to the 2D embedding and will interactively update coloring upon feature selection. To complete the portfolio, i2dash.scrnaseq provides functionality to display feature dynamics in an interactive heatmap (Figure 5B, use case 2 “SC data explorer”) by using the “heatmap” function with a SingleCellExperiment or Seurat object that contains features of interest.

**Figure 6:**
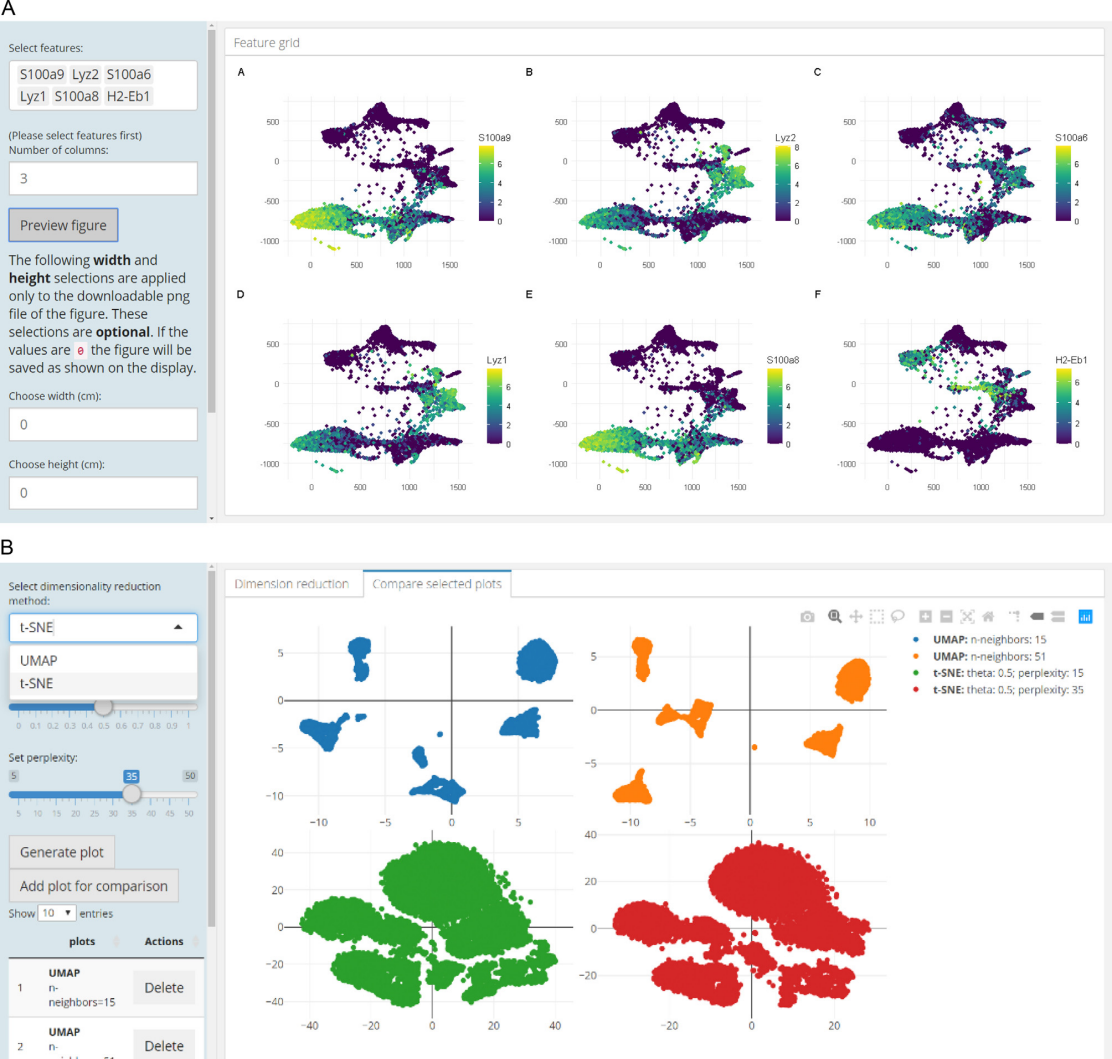
**Tool features of the i2dash.scrnaseq extension** **A.** A data view as provided by the “Feature grid page”, illustrating 2D embedded samples, colored by expression of arbitrary features. The number of grid columns and figure sizes can be customized, and the resulting figure, as well as the visualized data, are provided for download. **B.** The “DimRed comparison page” allows comparison of multiple 2D embeddings obtained by different runs of UMAP or t-SNE. Parameters can easily be defined using sliders, and results can be added individually to the comparison overview. t-SNE, t-distributed stochastic neighbor embedding; UMAP, uniform manifold approximation and projection.

Besides high-level functions for the visualization and interactive exploration of results from scRNA-seq analysis, i2dash.scrnaseq provides helpful tools to simplify otherwise cumbersome tasks. The “Feature grid page” can be used to interactively create publication-ready graphics that show a 2D embedding, colored by feature expression, for an arbitrary number of features on a grid layout (Figure 6A, use case 3 “Visual parameter selection for experts”) utilizing multipanelfigure [19]. In the context of dimension reduction methods, the “DimRed comparison page” simplifies the parameter search by comparing many different dimensionality reductions across different parameters (Figure 6B, use case 3 “Visual parameter selection for experts”). These data views are provided by the respective functions “add_feature_grid_page” and “add_dimred_comparison_page”, which once more rely on either a SingleCellExperiment or a Seurat object as input. Summing up, the i2dash extension i2dash.scrnaseq is an example for an individual view on a certain type of data (here scRNA-seq) that detaches the analysis of data (the package used for the analysis is open to the user) from the visualization of data as an interactive dashboard (coded in the i2dash.scrnaseq package).

## Benchmark and feature comparison

The R package i2dash is a generic tool for the generation of flexible dashboards. Therefore, it cannot be directly compared to specialized applications as enumerated above, dealing *e.g.*, only with SC specific aspects, such as iSEE does. However, by providing an exemplary i2dash extension for data from scRNA-seq experiments, a comprehensive comparison becomes feasible, emphasizing the generic approach of our framework. A detailed side-by-side comparison of web-based visualization tools from the perspective of implemented features is shown in Table 2. Briefly, all compared tools are R-based applications and are able to be deployed on a multitude of operating systems. In terms of general customization, i2dash offers a wider range of layouts and ways to organize content. While iSEE does allow a customized organization of visualization panels, i2dash extends this feature by organizing content via pages, menus, and sidebars. With regard to the content type, the other programs are limited to SC specific data types, provided as R objects. Only i2dash supports a variety of content types, including but not limited to images, text, and HTML widgets. Interactive and linked visualizations embedded in a Shiny app are provided by almost all programs. However, i2dash offers the unique possibility to link interactive visualizations in a customizable way, a feature of high importance when specific aspects of data should be prioritized (Table S2). Regarding the SC-related features, i2dash is capable of providing all key aspects of common scRNA-seq analysis. In summary, we conclude that i2dash and i2dash.scrnaseq at least substitute other specialized applications in the field of SC analysis and define a new standard for customizable, interactive, and web-based dashboards. Additionally, i2dash is easily extensible to match the functionality of existing tools and readily paves the way to large-scale cloud deployments.

**Table 2:** **Feature comparison between web-based applications focusing onscRNA-seq**

	**Category**	**Feature**	**i2dash + i2dash.scrnaseq**	**iSEE**	**Cerebro**	**pcaExplorer**	**scRNABatchQC**
General framework/dashboard functionality	Layout/Appearance	Grids	●	●	●	●	
		Colormaps	●	●	●		
		Tabset	●	○	○	●	
		Storyboard	●				
		Color themes	●				
	Content type	R objects	●	●	●		
		Images	●	○	●		
		Text/Markdown	●				
		htmlwidgets	●				
		HTML	●				
		Data Frames	●			●	●
	Content organization	Pages	●	●	●	●	
		Menus	●	●	●	●	
		Sidebars	●			●	
	Output	Shiny app	●	●	●	●	
		HTML website	●			●	●
	Visualization	Interactive plots	●	●	●	○	
		Linked interactive plots	●	●	●	●	
		Download data as spreadsheet	●		●	●	
	Portability	RMarkdown	●			●	
		Docker	●	●	●		
	Extensibility	Custom visualizations	●	●			
		Custom functions	●	●			
		Custom data types	●				

SC use case	Input	SingleCellExperiment	●	●			●
		Seurat	●		○*		●
	Quality control	Cell metadata	●	●	●	●	●*
		Gene metadata	●	●			
	Normalisation/ Batch correction		●			○	○*
	Feature selection		●*		●*		●*
	Dimension reduction	t-SNE	●*	●	●	○	●*
		UMAP	●*	●	●	○	
	Cell clustering		●	●	●		
	Marker genes		●	●	○*	○	○*

*Note*: ● indicates implemented feature/functionality; ○ indicates limited or in similar form; ●*/○* indicates calculated during the usage of the application. SC, single-cell; scRNA-seq, single-cell RNA sequencing; t-SNE, t-distributed stochastic neighbor embedding; UMAP, uniform manifold approximation and projection.

## Data availability

The latest stable version of i2dash can be installed from the comprehensive R archive network (CRAN, https://cran.r-project.org/package=i2dash), and the development version is available from https://github.com/loosolab/i2dash. We provide extensive documentation and vignettes, not only with the R package itself but also on a complementary website at https://loosolab.github.io/i2dash. Notably, we provide comprehensive tutorials addressing users at different experience levels. For beginners, a detailed guide on how to create an exemplary i2dash dashboard can be found at https://loosolab.github.io/i2dash/articles/i2dash-intro.html. For advanced users, we show how to extend the functionality of i2dash in order to fit dashboards to data types from other fields at https://loosolab.github.io/i2dash/articles/i2dash-extension.html. To facilitate automated deployments and dashboard development in isolated environments, thus reducing the burden of dependency management, we additionally provide i2dash as a Docker container, which can be obtained from https://gitlab.gwdg.de/loosolab/container/i2dash.deployment/container_registry/. Similarly, the i2dash extension i2dash.scrnaseq is available from https://github.com/loosolab/i2dash.scrnaseq. Use cases for application of i2dash.scrnaseq to data from scRNA-seq have been set up, and resulting dashboards and data views can be accessed from https://loosolab.github.io/i2dash.scrnaseq. Both packages have been tested on Linux, Windows, and MacOS.

## CRediT author statement

**Arsenij Ustjanzew:** Software, Methodology, Visualization, Writing - original draft. **Jens Preussner:** Conceptualization, Methodology, Software, Validation, Writing - original draft. **Mette Bentsen:** Methodology, Writing - review & editing. **Carsten Kuenne:** Methodology, Writing - review & editing. **Mario Looso:** Funding acquisition, Resources, Project administration, Supervision, Conceptualization, Writing - review & editing. All authors have read and approved the final manuscript.

## Competing interests

The authors have declared no competing interests.

## Supplementary material

Supplementary Table S1 **Component methods of the package i2dash.scrnaseq** A list of i2dash.scrnaseq’s high-level functions intended to generate complex plots.

Supplementary Table S2 **Predefined page methods of the package i2dash.scrnaseq** A list of i2dash.scrnaseq’s high-level functions intended to generate complex pages with predefined data views, including sets of dynamically linked plots.
