# Supplementary material for: i2dash: Creation of Flexible, Interactive, and Web-based Dashboards for Visualization of Omics Data
Source: Genomics Proteomics Bioinformatics. 2021 Jul 17;20(3):568–77. doi: 10.1016/j.gpb.2021.01.007 (PMC9801041; doi:10.1016/j.gpb.2021.01.007)
Supplement: Supplementary Table S1 — Component methods of the package i2dash.scrnaseq A list of i2dash.scrnaseq’s high-level functions intended to generate complex plots. [file mmc1.docx]

**Table S1 Component-methods of the package i2dash.scrnaseq**

| Method | Description |
| --- | --- |
| scatterplot | Renders a component containing a scatterplot with optional selection options. |
| violinplot | Renders a component containing a vertical violinplot with optional selection options. |
| boxplot | Renders a component containing a vertical boxplot with optional selection options. |
| barplot | Renders a component containing a horizontal barplot with optional selection options and two modes for absolute and relative visualisation. |
| heatmap | Renders a component containing a heatmap with optional selection options for aggregation, grouping, and clustering. |
| bubbleplot | Renders a component containing a bubble plot with optional selection options. |
| verbatim_text | Renders a component containing a verbatim text output element, which renders a reactive output. |
| summarize_metadata | Renders a component containing a table with summarized data. |
