# Supplementary material for: i2dash: Creation of Flexible, Interactive, and Web-based Dashboards for Visualization of Omics Data
Source: Genomics Proteomics Bioinformatics. 2021 Jul 17;20(3):568–77. doi: 10.1016/j.gpb.2021.01.007 (PMC9801041; doi:10.1016/j.gpb.2021.01.007)
Supplement: Supplementary Table S2 — Predefined page methods of the package i2dash.scrnaseq A list of i2dash.scrnaseq’s high-level functions intended to generate complex pages with predefined data views, including sets of dynamically linked plots. [file mmc2.docx]

**Table S2 Pre-defined page methods of the package i2dash.scrnaseq**

| Method | Description |
| --- | --- |
| add_dimred_feature_page | View a dimension reduction side-by-side with feature metadata. |
| add_feature_expression_page | Explore feature expression with dimension reductions, a violin plot, and a table grouped by sample metadata. |
| add_dimred_sample_page | Characterize and visualize dimension reductions and sample groupings/ metadata. |
| add_feature_grid_page | Create expression visualization for multiple selected features on a regular grid. |
| add_dimred_comparison_page | Explore the effects of the parameters "theta" and "perplexity" of a t-SNE or "n_neighors" of a UMAP embedding. |
| add_feature_selection_page | Quantify per-gene variation and explore the threshold on the metric of variation to get the desired set of highly variable features. |
